# AI-based automatic estimation of single-kidney glomerular filtration rate and split renal function using non-contrast CT

**DOI:** 10.1186/s13244-025-01959-x

**Published:** 2025-04-07

**Authors:** Yiwei Wang, Feng Xu, Qiuyue Han, Daoying Geng, Xin Gao, Bin Xu, Wei Xia

**Affiliations:** 1 Department of Urology, Ninth People’s Hospital, School of Medicine, Shanghai Jiao Tong University, Shanghai, China; 2 Department of Nuclear Medicine, Ninth People’s Hospital, School of Medicine, Shanghai Jiao Tong University, Shanghai, China; 3 Department of Radiology, Huashan Hospital, Fudan University, Shanghai, China; 4 Medical Imaging Department, Suzhou Institute of Biomedical Engineering and Technology, Chinese Academy of Sciences, Suzhou, China

**Keywords:** Renal function, Non-contrast CT, Deep learning, Radiomics

## Abstract

**Objectives:**

To address SPECT’s radioactivity, complexity, and costliness in measuring renal function, this study employs artificial intelligence (AI) with non-contrast CT to estimate single-kidney glomerular filtration rate (GFR) and split renal function (SRF).

**Methods:**

245 patients with atrophic kidney or hydronephrosis were included from two centers (Training set: 128 patients from Center I; Test set: 117 patients from Center II). The renal parenchyma and hydronephrosis regions in non-contrast CT were automatically segmented by deep learning. Radiomic features were extracted and combined with clinical characteristics using multivariable linear regression (MLR) to obtain a radiomics-clinical-estimated GFR (rcGFR). The relative contribution of single-kidney rcGFR to overall rcGFR, the percent renal parenchymal volume, and the percent renal hydronephrosis volume were combined by MLR to generate the estimation of SRF (rcphSRF). The Pearson correlation coefficient (*r*), mean absolute error (MAE), and Lin’s concordance coefficient (CCC) were calculated to evaluate the correlations, differences, and agreements between estimations and SPECT-based measurements, respectively.

**Results:**

Compared to manual segmentation, deep learning-based automatic segmentation could reduce the average segmentation time by 434.6 times to 3.4 s. Compared to single-kidney GFR measured by SPECT, the rcGFR had a significant correlation of *r* = 0.75 (*p* < 0.001), MAE of 10.66 mL/min/1.73 m^2^, and CCC of 0.70. Compared to SRF measured by SPECT, the rcphSRF had a significant correlation of *r* = 0.92 (*p* < 0.001), MAE of 7.87%, and CCC of 0.88.

**Conclusions:**

The non-contrast CT and AI methods are feasible to estimate single-kidney GFR and SRF in patients with atrophic kidney or hydronephrosis.

**Critical relevance statement:**

For patients with an atrophic kidney or hydronephrosis, non-contrast CT and artificial intelligence methods can be used to estimate single-kidney glomerular filtration rate and split renal function, which may minimize the radiation risk, enhance diagnostic efficiency, and reduce costs.

**Key Points:**

- Renal function can be assessed using non-contrast CT and AI.
- Estimated renal function significantly correlated with the SPECT-based measurements.
- The efficiency of renal function estimation can be refined by the proposed method.

**Graphical Abstract:**

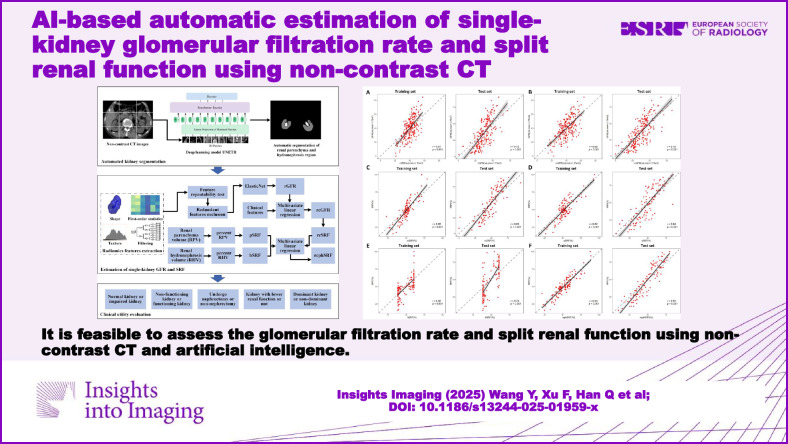

## Introduction

The glomerular filtration rate (GFR) is the best measure of kidney function [1]. The single-kidney GFR is required for preoperative evaluation in patients with atrophic kidney or hydronephrosis [2, 3]. The split renal function (SRF) represents the relative contribution of single-kidney GFR to overall GFR and is important for decision-making in urological surgery [4–6].

The single-photon emission computed tomography (SPECT) is the gold standard for measuring single-kidney GFR and SRF. Compared with SPECT, computed tomography (CT) is more commonly used due to its lower cost, faster imaging speed, and reduced radiation exposure. Previous investigations have used contrast-enhanced CT (CECT) to estimate renal function [7, 8]. However, the administration of iodine contrast media poses an elevated risk of nephrotoxicity [9, 10]. To avoid the use of iodinated contrast media, some studies found that the non-contrast CT-based renal parenchymal volume (RPV) is associated with single-kidney GFR [8, 11], and the percent RPV could be used to estimate SRF [5, 12, 13]. However, the RPV alone is insufficient for a comprehensive assessment of the health status of renal tissues, resulting in moderate performance. Besides, in previous studies, the calculation of RPV relied on the delineation of the renal parenchymal region, which is mainly achieved by labor-intensive manual segmentation, with limited potential for clinical application.

Artificial intelligence (AI) methods, including deep learning and radiomics, play an important role in medical image processing [14, 15]. Using deep learning algorithms, organ segmentation can be accomplished in an efficient, fully automated manner [16]. Besides, converting medical images into quantitative features through radiomics has great potential to extract more representative disease-related image features [14]. The renal parenchyma serves as the primary site for urine filtration, making its condition directly correlated with GFR. Furthermore, studies have indicated that increased severity of hydronephrosis is related to worse GFR [17, 18]. Therefore, we hypothesized that the radiomics features extracted from renal parenchyma and hydronephrosis regions could be used to estimate single-kidney GFR and SRF.

In this study, we aimed to develop and validate an AI-based automatic estimation method of single-kidney GFR and SRF using non-contrast CT for patients with hydronephrosis and atrophic kidney. Additionally, we assessed the clinical utility of estimations in distinguishing between kidneys with varying health statuses.

## Materials and methods

### Datasets and study design

Patients with atrophic kidney or hydronephrosis who underwent non-contrast CT within a 30-day period before or after SPECT imaging were included. An atrophic kidney is defined as one that has a length of less than 8 cm and is significantly smaller than the contralateral kidney, or where the renal parenchyma thickness is less than 1.5 cm. Hydronephrosis is defined as a condition where the anteroposterior diameter of the renal pelvis exceeds 10 mm.

The etiologies encompass ureteral calculi, ureteral strictures, or congenital anomalies of the ureteropelvic junction (UPJ). The exclusion criteria comprised of the following: (1) Renal tumors or cysts with a diameter exceeding 1 cm; (2) Kidney transplant, congenital or anatomical solitary kidney; (3) Anatomical variant of renal pelvis or calyces, such as ampulla renal pelvis; (4) Renal surgery before SPECT or CT imaging. Subsequently, a total of 128 patients from center I between Aug 2015 and Aug 2021 were included as the training set, while an independent test set consisting of 117 patients from center II between January 2017 and December 2019 was also included.

The non-contrast CT scan was conducted in center I using GE Discovery NM/CT 670 SPECT/CT scanner, with a tube voltage of 120 V and tube current of 180 mA. The slice thickness was set to 5 mm, and the matrix was configured to be 512 × 512. The non-contrast CT of center II was performed using Canon Aqulion 64-slice helical CT scanner with tube voltage of 120 V, tube current of 230 mA, slice thickness of 5 mm and matrix size of 512 × 512.

The SPECT imaging was performed using GE Discovery NM/CT 670 SPECT/CT scanner in Center I and GE Infinia SPECT in Center II. The single-kidney GFR was measured through SPECT dynamic imaging with ^99^mTc-DTPA [8]. The same imaging parameters were applied in both center I and center II, including matrix size of 128 × 128, magnification of 1.0, collection time for each frame set at 15 s with a total of 60 frames collected. Following the examination, original data underwent processing by the workstation to obtain the time-radioactivity curve and the single-kidney GFR values were determined utilizing the Gates method. Subsequently, SRF was calculated as the relative contribution of single-kidney GFR to overall GFR.

**Fig. 1:**
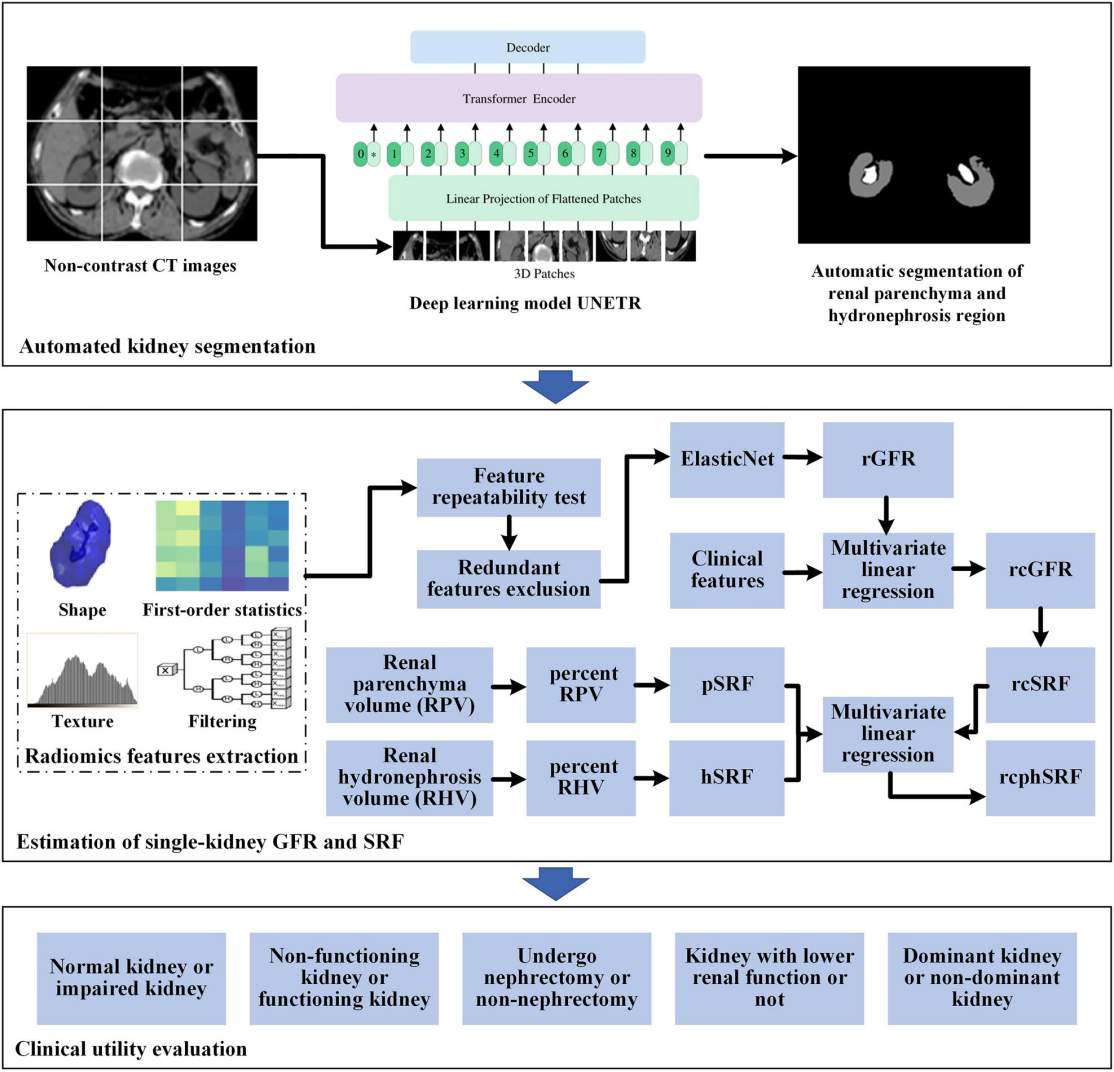
The workflow of this study. A deep learning model UNETR was trained to perform automatic segmentation of the renal parenchyma and hydronephrosis regions in non-contrast CT images. Then, radiomic features were extracted from these regions to estimate single-kidney GFR (rGFR). The rGFR was further refined as rcGFR by combining with clinical features. Then, the rcGFR, the renal parenchyma and hydronephrosis volumes were used to estimate SRF (rcphSRF). Finally, the clinical utility of rcGFR and rcphSRF in distinguishing between kidneys with varying health statuses was evaluated

The study received approval from the Institutional Review Board of all participating centers, and due to its retrospective nature, the requirement for informed consent was waived. Figure 1 illustrates the workflow of this multicenter study.

### Automated segmentation of renal parenchyma and hydronephrosis

The renal parenchyma and hydronephrosis regions were manually delineated in each kidney by two radiologists with 10 years of experience using the MITK software (version 2018.04) and confirmed by a radiologist with 25 years of experience. The renal pelvis, sinusoidal fat, and visible major vessels (renal artery and vein) in the hilum were excluded when delineating the renal parenchyma region on non-contrast CT. The hydronephrosis region was defined as the renal pelvis area with water-like density. The voxel intensity range of non-contrast CT images was clipped into the kidney window (width = 260 HU, level = 60 HU) and normalized using the z-score method.

All 3D non-contrast CT images were resampled to a size of 256 × 256 × 80. Next, the delineations and 3D non-contrast CT images were used to train the 3D segmentation model UNETR [19] for automatic segmentation of renal parenchyma and hydronephrosis. UNETR maintains the U-shaped structure of the U-Net [20] while using the transformer [21] as the encoder, and it has the advantage of extracting image features at different scales, which is suitable for segmenting the renal parenchyma and hydronephrosis regions with different sizes. The UNETR was trained using Python 3.9, PyTorch 1.9 in a workstation with NVIDIA GeForce RTX 3090. The parameters were as follows: max epoch, 1000; loss function, diceloss; optimizer, adamw; learning rate, 0.0001. Data augmentation, including random cropping, flipping and rotation, was performed during model training.

### Estimation of GFR and SRF

For both manual segmentation and automated segmentation, we extracted 2260 radiomic features from the 3D non-contrast CT volume of renal parenchyma and hydronephrosis regions using PyRadiomics, including 28 shape features, 36 first-order statistic features, 150 texture features and 2046 filtering features. The shape features, first-order statistic features, and texture features were extracted from original images, while the filtering features were extracted from the wavelet, square, logarithm, and gradient filtered images [22].

To improve the repeatability of the radiomic features, features with an intraclass coefficient (ICC) between manual and automatic segmentation below 0.9 were excluded. In addition, we calculated pairwise feature Spearman correlation coefficients (SCCs), and the feature pairs with an SCC > 0.9 were identified as highly correlated. In each highly correlated feature pair, the SCCs between a feature and all the other features were calculated, and the feature with the larger mean SCC was considered redundant and excluded. The remaining features were input into the ElasticNet [23] to generate radiomics-estimated GFR (rGFR). The ElasticNet could select the most representative features and determine the weights of the features to estimate the single-kidney GFR. The hyperparameters of ElasticNet were determined by ten-fold cross-validation. The rGFR was further combined with clinical characteristics using multivariate linear regression (MLR) to obtain a more accurate radiomics-clinical-estimated GFR (rcGFR).

Then, four estimations of SRF were calculated: (1) rcSRF was calculated as the relative contribution of single-kidney rcGFR to overall rcGFR; (2) pSRF was calculated using the percent RPV with linear regression [5, 24–26]; (3) hSRF was calculated using the percent renal hydronephrosis volume (RHV) with linear regression; (4) rcphSRF was calculated by combining the rcSRF, pSRF, and hSRF with MLR.

### Statistical analysis

The baseline clinical characteristics of training and test set were compared using the Mann–Whitney U test (for continuous parameters) and χ^2^ test (for categorical variables). The performance of automatic segmentation of renal parenchyma and hydronephrosis was evaluated using the Dice similarity coefficient (DSC) with manual segmentation as the ground truth. The agreements and differences between model predictions and the SPECT-based measurements were investigated using the slope and intercept of the reduced major axis, Lin’s concordance coefficient (CCC), the mean absolute error (MAE), mean squared error (MSE), and the Bland-Altman plot with Limits of Agreement (LoA). Pearson correlation analysis and paired-sample *t*-tests between true and predicted values were also reported. Wilcoxon signed-rank test was used to compare the MAE and MSE between estimations based on features extracted from manual and automatic segmentation.

To evaluate the clinical value of estimations, previously reported cutoffs were used to distinguish the kidneys with different health statuses [5, 24, 27–31]: (1) impaired kidney (single-kidney GFR ≤ 30 mL/min × 1.73 m^2^) or normal kidney (single-kidney GFR > 30 mL/min × 1.73 m^2^); (2) non-functioning kidney (single-kidney GFR < 10 mL/min × 1.73 m^2^) or functioning kidney (single-kidney GFR ≥ 10 mL/min × 1.73 m^2^); (3) undergo nephrectomy (SRF < 15%) or non-nephrectomy (SRF ≥ 15%); (4) kidney with lower renal function (SRF < 45%) or not (SRF ≥ 45%); (5) non-dominant kidney (SRF ≤ 60%) or dominant kidney (SRF > 60%). The area under the receiver operating characteristic curve (AUC), accuracy, sensitivity, and specificity of estimations in discriminating the kidneys with different health statuses were calculated. Delong test was used to compare the AUC between estimations based on features extracted from manual and automatic segmentation.

The modeling and statistical analysis were performed using R (version 3.6.1). A *p*-value < 0.05 was considered statistically significant.

## Results

### Characteristics of the patient cohort

The baseline characteristics of patients are presented in Table 1. The characteristics were compared between training set and test set, and there were no significant differences except for RPV and single-kidney GFR.

**Table 1:** Clinical characteristics of the patients

Characteristics	Training set	Test set	*p*-value
Gender			0.853
Male	74 (57.8)	69 (59.0)	
Female	54 (42.2)	48 (41.0)	
Age (years)	52.76 ± 13.70	55.79 ± 16.39	0.070
BMI	23.90 ± 3.29	23.86 ± 3.23	
RPV (mL)	152.83 ± 51.58	132.70 ± 60.43	< 0.01
RHV (mL)	34.51 ± 67.98	47.51 ± 91.18	0.408
Single-kidney GFR (mL/min × 1.73 m^2^)	40.36 ± 14.43	36.27 ± 19.41	0.002

For gender, the data are numbers of patients, and data in parentheses are percentages. Age, BMI, RPV, RHV, and single-kidney GFR are mean ± standard deviations

The *p*-values were derived from the Mann–Whitney U test (for continuous parameters) and χ^2^ test (for categorical variables)

*BMI* body mass index, *RPV* renal parenchyma volume, *RHV* renal hydronephrosis volume, *GFR* glomerular filtration rate

### Performance of automatic segmentation of renal parenchyma and hydronephrosis

**Fig. 2:**
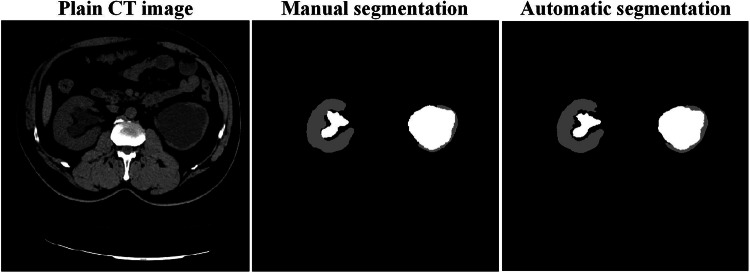
Example of automatic kidney segmentation. The regions of renal parenchyma and renal hydronephrosis are respectively annotated in shades of gray and white

Using manual segmentation (average segmentation time of 1477.9 s per case) as the reference standard, the mean DSC of automatic renal parenchyma segmentation was 0.893 ± 0.105 in the training set and 0.844 ± 0.132 in the test set, and the mean DSC of automatic renal hydronephrosis segmentation was 0.732 ± 0.323 in the training set and 0.686 ± 0.305 in the test set. The average processing time of segmentation was only 3.4 s per case. The representative results of automatic segmentation are illustrated in Fig. 2.

### The estimation of GFR

Based on the results of automatic segmentation, a total of 2260 radiomic features were extracted from the regions of renal parenchyma and renal hydronephrosis in CT images. After eliminating low-repeatability and highly redundant features, 33 features were retained. The ElasticNet ultimately identified 5 features from the 33 features to generate the formula of rGFR (Table S1), including four features from the renal parenchyma region and one feature from the renal hydronephrosis region.

**Fig. 3:**
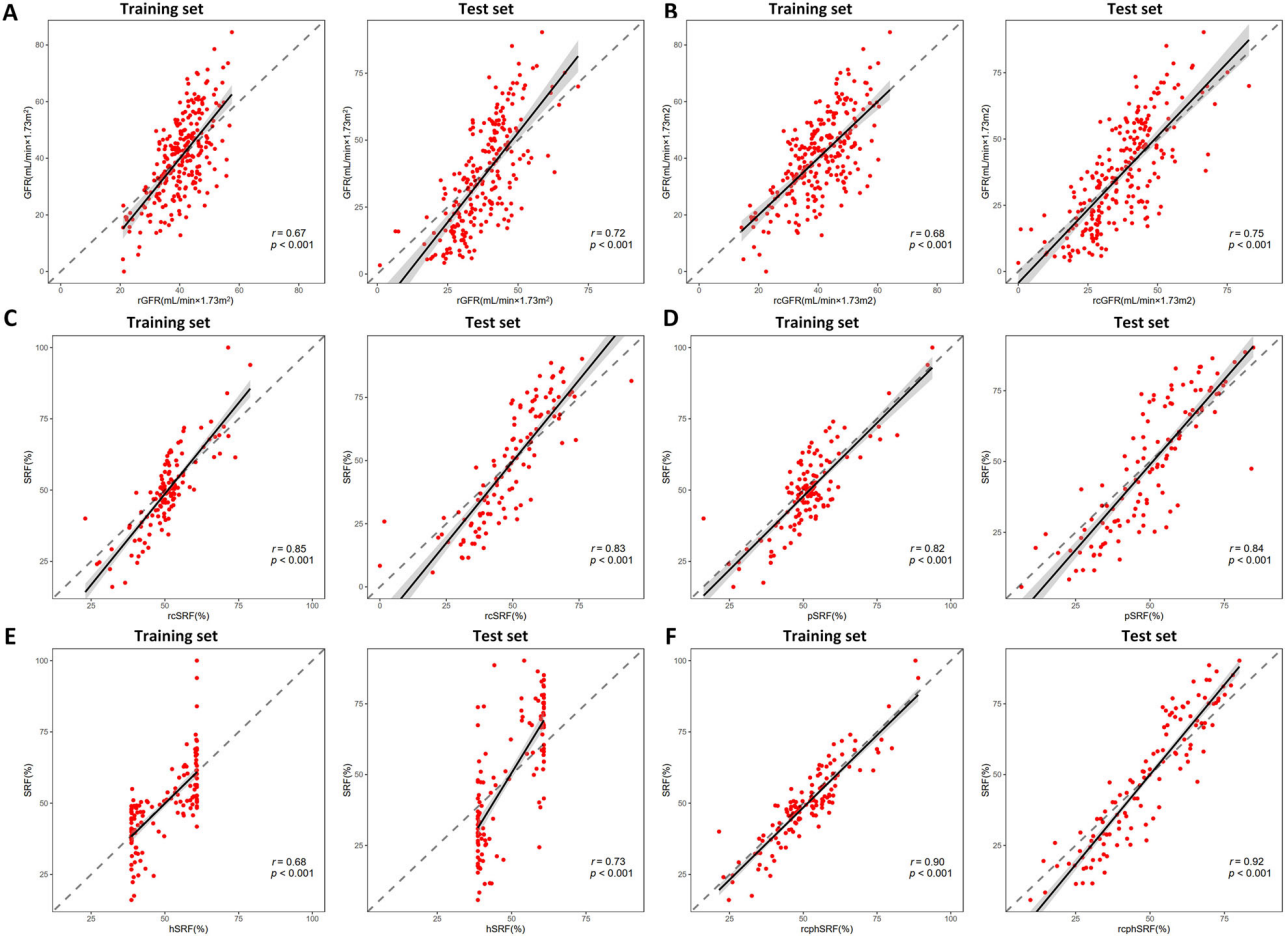
The correlation plots of estimations and SPECT-based measurement. The dashed lines indicate perfect agreement. **A** rGFR. **B** rcGFR. **C** rcSRF. **D** pSRF. **E** hSRF. **F** rcphSRF

The correlation between rGFR and SPECT-measured GFR is presented in Table 2 and Fig. 3. The Pearson correlation analysis indicated that rGFR was correlated well with SPECT-measured GFR (*r* = 0.72, *p* < 0.001). The paired-sample *t*-test showed that there were no significant differences between the rGFR and SPECT-measured GFR (*p* > 0.05). Meanwhile, the rGFR had a relatively poor CCC of 0.60.

**Table 2:** Comparisons of estimations and SPECT-based measurements

Estimation	*r*	*p*-value^§^	MAE	MSE	*p*-value*	CCC	Slope	Intercept
rGFR								
Training set	0.67	< 0.001	8.76	119.20	0.989	0.55 (0.48, 0.61)	1.93	−37.52
Test set	0.72	< 0.001	11.74	195.11	0.085	0.60 (0.54, 0.66)	1.85	−33.68
rcGFR								
Training set	0.68	< 0.001	8.31	110.67	0.762	0.64 (0.57, 0.70)	1.72	−18.70
Test set	0.75	< 0.001	10.66	164.07	0.669	0.70 (0.64, 0.75)	1.46	−17.53
rcSRF								
Training set	0.85	< 0.001	6.06	61.99	0.011	0.77 (0.73, 0.81)	1.51	−0.26
Test set	0.83	< 0.001	10.65	162.32	0.314	0.78 (0.74, 0.81)	1.52	−0.26
pSRF								
Training set	0.82	< 0.001	6.51	65.21	< 0.001	0.79 (0.74, 0.83)	1.24	−0.12
Test set	0.84	< 0.001	10.09	158.91	0.212	0.79 (0.75, 0.83)	1.43	−0.22
hSRF								
Training set	0.68	< 0.001	7.66	98.47	0.801	0.64 (0.57, 0.70)	1.46	−2.28
Test set	0.73	< 0.001	13.89	281.95	0.507	0.53 (0.48, 0.59)	2.32	−0.66
rcphSRF								
Training set	0.90	< 0.001	4.92	37.82	< 0.001	0.89 (0.86, 0.91)	1.12	−0.06
Test set	0.92	< 0.001	7.87	93.45	0.406	0.88 (0.86, 0.90)	1.37	−0.18

*r* is the Pearson correlation coefficient. The slope and intercept were derived from the reduced major axis regression. CCC is Lin’s concordance coefficient with 95% confidence interval in parentheses. rcSRF was calculated as the relative contribution of single-kidney rcGFR to overall rcGFR, pSRF was estimated based on the percentage of renal parenchymal volume (RPV), hSRF was estimated based on the percentage of renal hydronephrosis volume (RHV), rcphSRF was derived from the multivariate linear regression of rcSRF, pSRF, and hSRF. The unit of GFR is mL/min × 1.73 m^2^, and the unit of SRF is %

*MAE* mean absolute error, *MSE* mean squared error, *GFR* glomerular filtration rate, *SRF* split renal function, *rGFR* radiomics-estimated GFR, *rcGFR* radiomics-clinical-estimated GFR

§ *p*-value was derived from Pearson correlation analysis

* *p*-value was derived from paired-sample *t*-test

The multivariate linear regression revealed that age and rGFR were independent predictors, and the rcGFR was calculated as the linear combination of age and rGFR (Table S2). Compared to rGFR, rcGFR demonstrated stronger correlations (*r* = 0.75 vs. 0.72), less MAE (10.66 vs. 11.74) and MSE (164.07 vs. 195.11) in test set, along with higher CCC values (0.70 vs. 0.60). Furthermore, rcGFR exhibited a reduced slope (1.46 vs. 1.85) and an increased intercept (−17.53 vs. −33.68), bringing them closer to perfect agreement (slope = 1 and intercept = 0). These findings indicate that rcGFR possesses superior predictive capabilities compared to rGFR.

**Fig. 4:**
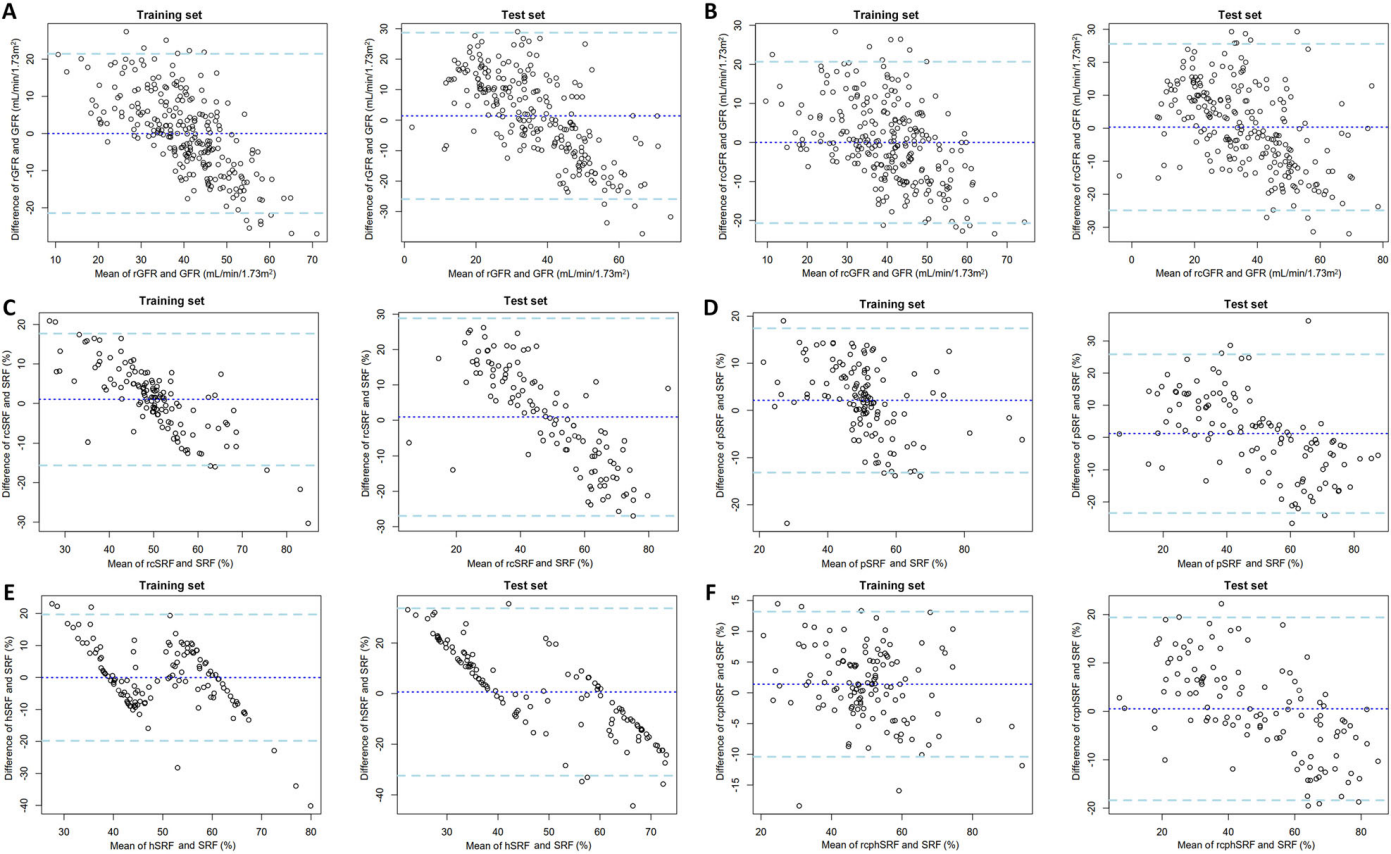
The Bland-Altman plots of estimations and SPECT-based measurements. The 95% limits of agreement (LoA) were between the dashed lines. The dotted lines were the mean differences. **A** rGFR. **B** rcGFR. **C** rcSRF. **D** pSRF. **E** hSRF. **F** rcphSRF

Besides, the Bland-Altman plot in Fig. 4 confirmed that most of the differences between the estimated GFRs and SPECT-measured GFR were within the LoA, indicating good consistency.

For the estimation of GFR (Table S4), no significant differences in error were observed between manual and automatic segmentation-based models. These estimations relied on robust radiomics features, with unstable features—defined by an ICC below 0.9 between manual and automatic segmentations—being excluded from the analysis.

### The estimation of SRF

The rcSRF was derived from rcGFR, and exhibited a robust correlation with SPECT-measured SRF (*r* = 0.83, *p* < 0.001), along with a moderate CCC of 0.78. Compared to rcSRF, the pSRF demonstrated similar correlation (*r* = 0.84 vs. 0.83) and CCC (0.79 vs. 0.78), with comparable slope (1.43 vs. 1.52) and intercept (−0.26 vs. −0.22), but smaller MAE (10.09 vs. 10.65) and MSE (158.91 vs. 162.32). On the other hand, hSRF exhibited the weakest correlation (*r* = 0.73, *p* < 0.001), the lowest CCC of 0.53, and had the largest MAE of 13.89 and MSE of 281.95.

The rcSRF, pSRF, and hSRF were identified as independent predictors of SRF through multivariate linear regression and subsequently combined to form a composite estimation rcphSRF (Table S3). In the test set, rcphSRF achieved the strongest correlation (*r* = 0.92, *p* < 0.001), the smallest MAE of 7.87 and MSE of 93.45, and the highest CCC of 0.88. Additionally, rcphSRF exhibited the closest slope (1.37) and intercept (−0.18) to the perfect agreement. These findings confirm that rcphSRF had the best predictive ability among other estimations.

The Bland-Altman plot further confirmed that over 90% of the differences between the estimated SRFs and SPECT-measured SRFs were within the LoA, indicating good consistency.

For the estimation of SRF (Table S4), significant discrepancies in error were noted between models based on manual segmentation versus those based on automatic segmentation. This discrepancy primarily stems from the reliance on the volumes of renal parenchyma and hydronephrosis regions (pSRF and hSRF), where the performance of automatic segmentation remains suboptimal.

### Performance of estimations for discriminating kidneys with different health statuses

The discriminative performance of rcGFR and rcphSRF for kidneys with different health statuses was calculated and presented in Table 3. The values of performances in discriminating impaired or normal and non-functioning or functioning kidneys pertained to rcGFR, and other values of performances pertained to rcphSRF. Overall, both rcGFR and rcphSRF demonstrated high AUC, accuracy, and specificity, but relatively low sensitivity, particularly in identifying non-functioning kidneys or those requiring nephrectomy. In discriminating between impaired or normal, lower renal function or not, non-dominant or dominant kidneys, the performances of rcGFR and rcphSRF were relatively balanced.

**Table 3:** The performance of rcGFR and rcphSRF in discriminating the kidneys with varying health status

Health status	AUC (95% CI)	Accuracy (95% CI)	Sensitivity (95% CI)	Specificity (95% CI)
Impaired or normal				
Training set	0.840 (0.779, 0.901)	85.2 [218/256] (80.2, 89.3)	46.7 [28/60] (33.7, 60.0)	96.9 [190/196] (93.5, 98.9)
Test set	0.862 (0.813, 0.910)	81.2 [190/234] (75.6, 86.0)	71.4 [70/98] (61.4, 80.1)	88.2 [120/136] (81.6, 93.1)
Non-functioning or functioning				
Training set	0.977 (0.956, 0.998)	98.4 [252/256] (96.0, 99.6)	0 [0/4] (0, 60.2)	100 [252/252] (98.5, 100)
Test set	0.911 (0.856, 0.965)	91.0 [213/234] (86.6, 94.4)	10.5 [2/19] (1.3, 33.1)	98.1 [211/215] (95.3, 99.5)
Nephrectomy or non-nephrectomy				
Training set	1.000 (1.000, 1.000)	100 [256/256] (98.6, 100)	100 [2/2] (15.8, 100)	100 [254/254] (98.6, 100)
Test set	0.959 (0.929, 0.989)	97.0 [227/234] (93.9, 98.8)	22.2 [2/9] (2.8, 60.0)	100 [225/225] (98.4, 100)
Lower renal function or not				
Training set	0.938 (0.910, 0.967)	86.7 [222/256] (81.9, 90.6)	80.0 [60/75] (69.2, 88.4)	89.5 [162/181] (84.1, 93.6)
Test set	0.980 (0.966, 0.994)	92.3 [216/234] (88.1, 95.4)	88.2 [90/102] (80.4, 93.8)	95.5 [126/132] (90.4, 98.3)
Non-dominant or dominant				
Training set	0.958 (0.934, 0.982)	90.6 [222/256] (86.4, 93.9)	96.6 [198/205] (93.1, 98.6)	66.7 [34/51] (52.1, 79.2)
Test set	0.964 (0.944, 0.983)	87.2 [204/234] (82.2, 91.2)	93.2 [136/146] (87.8, 96.7)	77.3 [68/88] (67.1, 85.5)
Mean				
Training set	0.943	92.2	64.7	90.6
Test set	0.935	89.7	57.1	91.8

Data are percentages, with numbers of kidneys in square brackets and 95% confidence interval in parentheses. The kidneys with poor health status (i.e., impaired, non-functioning, undergo nephrectomy, lower renal function, or non-dominant) are considered as positive to calculate the sensitivity and specificity. The values of performances in discriminating impaired or normal and non-functioning or functioning kidneys pertained to rcGFR, and other values of performances pertained to rcphSRF

For all tasks related to discriminating kidney health statuses, no significant differences in AUC were observed between rcGFR and rcphSRF estimations based on features extracted from both manual and automatic segmentation (Table S5). Since these tasks rely on predefined cutoff values, they are relatively insensitive to errors in continuous estimation values.

## Discussion

Our findings suggest that the deep learning model was able to automatically segment renal parenchyma and renal hydronephrosis regions in non-contrast CT. The radiomic features extracted from the two regions could be used to estimate single-kidney GFR and SRF. The estimations exhibited high accuracy and specificity in discriminating kidneys with varying health statuses. Despite differences in patient characteristics between the training and test sets, the models demonstrated good performance in both datasets. This suggests that the models have good generalizability across different patient populations.

Previous studies reported that CECT can be used to assess the renal function [7, 8]. However, the administration of contrast medium during CECT may impair renal function and is contraindicated in patients with deteriorating renal function or a history of contrast media allergy [5]. Subsequently, several studies attempted to estimate single-kidney GFR and SRF based on non-contrast CT and reported that the RPV and the percent RPV were related to GFR and SRF [5, 11–13]. However, the RPV alone is insufficient [8] for a comprehensive assessment of the health status of renal tissues, resulting in moderate performance. Besides, they generally included small cohorts from a single center, and the generalization needs further verification. Furthermore, the calculation of RPV relied on labor-intensive manual kidney segmentation (about 15 min per patient [12]), which limited its clinical application.

To the best of our knowledge, this is the first attempt to use AI to assess the renal function based on non-contrast CT. Our results demonstrated that the deep learning model can achieve automatic segmentation of renal parenchyma and hydronephrosis regions, reducing the average segmentation time by 434.6 times to 3.4 s, thereby making the segmentation-based renal function estimation clinically applicable.

Moreover, non-contrast CT-based radiomic features could be used to estimate single-kidney GFR as rGFR. Regarding the radiomics features used in calculating rGFR, most of them (4/5) were from the renal parenchyma region, this was consistent with the fact that the renal parenchyma is the primary site for urine filtration, making its condition directly correlated with GFR. For features of renal parenchyma, the coefficients were all positive, which means that the larger the feature values, the larger the rGFR. Higher LargeAreaHighGrayLevelEmphasis and TotalEnergy values may indicate a large and intrinsically high-density area in the renal parenchyma. This can be associated with less edema or fibrosis, as these pathological conditions often reduce the CT values of tissue. Healthy renal parenchyma generally has higher tissue density, which means better structural integrity and a lower degree of pathological changes, which may indicate better renal function. In addition, higher SizeZoneNonUniformity and Busyness values on CT may reflect the complexity and diversity of different tissue structures within the renal parenchyma, it may represent normal differences between the glomeruli and tubule that are necessary to maintain normal nephron function such as filtration and reabsorption. For renal hydronephrosis region, the coefficient of the feature Busyness was negative, which means that the larger the Busyness, the smaller the rGFR. A higher value of Busyness reflected the larger changes in CT values, and it may suggest the more complex composition of the hydronephrosis, which indicated more severe and prolonged hydronephrosis and worse renal function. Furthermore, age was another independent predictor of GFR, and this was consisted with previous findings that the age-related changes in the renal structure can lead to a gradual decrease in renal function [32]. Moreover, age could complement rGFR to achieve a better estimation (rcGFR).

The rcGFR was further used to calculate a novel SRF estimation rcSRF. The rcSRF, pSRF, and hSRF were identified as independent predictors of SRF. The non-contrast CT-based rcSRF and pSRF exhibited similar robust correlations with SPECT-based measurement, and they had stronger correlations than the hSRF. Meanwhile, these predictors can be combined as a better estimation rcphSRF than separately, indicating that different estimations are complementary to reduce errors. In contrast, previous studies only focused on pSRF [5, 12, 13].

Few studies explored the clinical usefulness of estimations, while this study examined the accuracy, sensitivity, and specificity of rcGFR and rcphSRF in distinguishing between kidneys with varying health statuses. Overall, both rcGFR and rcphSRF demonstrated high performance. However, the overall sensitivity was relatively low, possibly due to the imbalanced sample distribution in real clinical settings where the number of kidneys with poor health status was less than healthy kidneys, particularly for those kidneys requiring nephrectomy or non-functioning kidneys.

Our study had several limitations. First, the use of non-contrast CT with 5 mm slice thickness may affect the extraction of the radiomic features due to partial volume effect, while the use of non-contrast CT with 1 mm slice thickness may alleviate the effect to improve the estimation performance. Second, the blood urea nitrogen and blood creatinine measurements were absent in the included cohorts, as the primary objective of our study was to investigate the feasibility of using non-contrast CT scans combined with AI to estimate single-kidney GFR and SRF. Future prospective studies were desired to explore whether incorporating these blood markers can further optimize the models. Third, for the estimation of SRF, there were significant differences in error between models based on manual segmentation versus those based on automatic segmentation. This highlights a limitation, underscoring the necessity for future research aimed at enhancing the accuracy of automatic segmentation for both renal parenchyma and hydronephrosis.

In conclusion, the utilization of non-contrast CT and AI techniques enabled automatic estimation of single-kidney GFR and SRF in patients with atrophic kidney or hydronephrosis. Our approach showed great potential in effectively minimizing radiation exposure, enhancing estimation efficiency, and reducing associated costs.

## Supplementary information

ELECTRONIC SUPPLEMENTARY MATERIAL

## Data Availability

The datasets generated and analyzed during the current study are not publicly available but are available from the corresponding author on reasonable request.

## Abbreviations

AI: Artificial intelligence
AUC: Area under the receiver operating characteristic curve
CECT: Contrast-enhanced CT
DSC: Dice similarity coefficient
GFR: Glomerular filtration rate
ICC: Intraclass coefficient
MLR: Multivariable logistic regression
SCCs: Spearman correlation coefficients
SPECT: Single-photon emission computed tomography
SRF: Split renal function
