# Supplementary material for: AI-based automatic estimation of single-kidney glomerular filtration rate and split renal function using non-contrast CT
Source: Insights Imaging. 2025 Apr 7;16:84. doi: 10.1186/s13244-025-01959-x (PMC11977085; doi:10.1186/s13244-025-01959-x)
Supplement: Supplementary file 1 — ELECTRONIC SUPPLEMENTARY MATERIAL [file 13244_2025_1959_MOESM1_ESM.pdf]

AI-based automatic estimation of single-kidney glomerular filtration rate and split renal function using non-contrast CT
ELECTRONIC SUPPLEMENTARY MATERIAL

Supplement A: Details of estimated GFR and SRF

The rGFR was calculated as the weighted sum of radiomics features (Table S1). The rcGFR was the weighted sum of rGFR and age (Table S2). The pSRF was caculated as 0.991×percent RPV+0.5%, and the hSRF was calculated as -0.223×percent RHV+61.1%. The rcphSRF was the weighted sum of the rcSRF, pSRF, and hSRF (Table S3). The coefficients of features and variables were listed in the following Tables.

Table S1. The coefficients of radiomics features in the formula of calculating rGFR.

Features and intercept	Coefficients
p_original_glszm_LargeAreaHighGrayLevelEmphasis	3.592×10^{-7}
p_original_glszm_SizeZoneNonUniformity	2.147×10^{-5}
p_gradient_ngtdm_Busyness	2.880
p_square_firstorder_TotalEnergy	1.353×10^{-8}
h_square_ngtdm_Busyness	-0.1498
Intercept	19.585

Note.—The features with ‘p_’ were extracted from the renal parenchyma region, while those with ‘h_’ were extracted from the renal hydronephrosis region. rGFR=radiomics-estimated GFR. GFR=glomerular filtration rate.

Table S2. The multivariate linear regression analysis of rcGFR.

	Full multivariable model		Final model of rcGFR	
Variables and intercept	Coefficients	*P*	Coefficients	*P*
Age (years)	-0.145	0.007	-0.147	0.004
Gender (Male *vs.* female)	-1.817	0.197	-	-
BMI (Abnormal *vs.* normal)	-1.858	0.199	-	-
RPV (ml)	-6.485×10^{-6}	0.920	-	-
RHV (ml)	-3.413×10^{-5}	0.262	-	-
rGFR (mL/min×1.73m^2)	1.203	<0.001	1.224	<0.001
Intercept	2.265	0.691	-1.303	0.799

Note.—rcGFR=radiomics-clinical-estimated GFR, GFR=glomerular filtration rate, BMI=Body mass index, RPV=renal parenchyma volume, RHV=renal hydronephrosis volume.

Table S3. The multivariate linear regression analysis of rcphSRF.

Variables and intercept	Coefficients	*P*
rcSRF (%)	0.416	0.009
pSRF (%)	0.564	<0.001
hSRF (%)	0.535	<0.001
Intercept (%)	-25.8	<0.001

Note.—SRF=split renal function. rcSRF was calculated as the relative contribution of single-kidney rcGFR to overall rcGFR, pSRF was estimated by percent renal parenchymal volume (RPV), hSRF was estimated by percent renal hydronephrosis volume (RHV), rcphSRF was the weighted sum of the rcSRF, pSRF, and hSRF. rcGFR=radiomics-clinical-estimated GFR.

Table S4. The MAE and MSE of estimations based on features extracted from manual and automatic segmentation.

Estimation	MAE (Manual segmentation)	MAE (Automatic segmentation)	*p*	MSE (Manual segmentation)	MSE (Automatic segmentation)	*p*
**rGFR**						
Training set	9.03	8.76	0.108	124.00	119.20	0.089
Test set	11.52	11.74	0.287	188.58	195.11	0.635
**rcGFR**						
Training set	8.51	8.31	0.271	113.43	110.67	0.409
Test set	10.34	10.66	0.419	153.70	164.07	0.550
**rcSRF**						
Training set	5.86	6.06	0.773	57.56	61.99	0.766
Test set	10.07	10.65	0.101	142.45	162.32	0.018
**pSRF**						
Training set	6.05	6.51	0.008	54.86	65.21	0.006
Test set	7.68	10.09	<0.001	94.86	158.91	<0.001
**hSRF**						
Training set	7.94	7.66	0.315	118.45	98.47	0.113
Test set	14.34	13.89	<0.001	300.52	281.95	<0.001
**rcphSRF**						
Training set	4.96	4.92	0.567	35.69	37.82	0.635
Test set	6.43	7.87	<0.001	64.57	93.45	<0.001

Note.- *p* values were derived from Wilcoxon signed-rank test. MAE=mean absolute error, MSE=mean squared error. The unit of GFR is mL/min×1.73m^2, and the unit of SRF is %.

Table S5. The AUC of rcGFR and rcphSRF based on features extracted from manual and automatic segmentation in discriminating the kidneys with varying health status.

Health status	AUC (Manual segmentation)	AUC (Automatic segmentation)	*p*
**Impaired or normal**			
Training set	0.841 (0.783, 0.900)	0.840 (0.779, 0.901)	0.838
Test set	0.870 (0.823, 0.916)	0.862 (0.813, 0.910)	0.511
**Non-functioning or functioning**			
Training set	0.987 (0.970, 1.000)	0.977 (0.956, 0.998)	0.085
Test set	0.916 (0.860, 0.971)	0.911 (0.856, 0.965)	0.484
**Nephrectomy or non-nephrectomy**			
Training set	1.000 (1.000, 1.000)	1.000 (1.000, 1.000)	1.000
Test set	0.976 (0.948, 1.000)	0.959 (0.929, 0.989)	0.195
**Lower renal function or not**			
Training set	0.934 (0.905, 0.963)	0.938 (0.910, 0.967)	0.561
Test set	0.982 (0.970, 0.995)	0.980 (0.966, 0.994)	0.493
**Non-dominant or dominant**			
Training set	0.962 (0.940, 0.985)	0.958 (0.934, 0.982)	0.521
Test set	0.968 (0.950, 0.986)	0.964 (0.944, 0.983)	0.440
**Mean**			
Training set	0.945	0.943	0.957
Test set	0.942	0.935	0.820

Note.- For each task, *p* values were derived from Delong test. For mean value, *p* values were derived from t test. Data in parentheses are 95% confidence interval. The values of performances in discriminating impaired or normal and non-functioning or functioning kidneys pertained to rcGFR, and other values of performances pertained to rcphSRF.
